# Ubiquitous microbial contaminants associated with scientific ocean drilling

**DOI:** 10.1099/acmi.0.000865.v3

**Published:** 2025-03-07

**Authors:** Jessica M. Labonté, Kathryn L. Campbell, Jordan R. Walker, Milena A. Rodriguez-Pilco

**Affiliations:** 1 Department of Marine Biology, Texas A&M University at Galveston, Galveston, TX, 77554, USA

**Keywords:** 16S rRNA gene sequencing, deep-sea sediments, geomicrobiology, microbial contamination, scientific ocean drilling

## Abstract

Deep-sea subsurface samples typically have low microbial biomass, making them more susceptible to contamination. Potential contaminants can be introduced during any step of the scientific process, including drilling (contamination from the drilling fluid and lubricants and seawater); sample preparation (contamination from air and human handling); and DNA extraction, amplification and sequencing (contamination from reagents). The International Ocean Discovery Program (IODP) samples that are dedicated to microbiological analyses (known as MBIO samples) are routinely tested for contamination by injecting known concentrations of fluorescent microspheres or tracers directly into the drilling fluids. These tracers are a great tool to determine the level of drilling fluid contamination on board, but they are not consistently used, and contamination of the samples could occur during other steps of sample processing and analysis. Moreover, there is an increased risk of contamination dominating the results of microbial surveys using PCR amplification of marker genes. Here, we built a database of common contaminants through the screening of contamination controls from available 16S rRNA gene amplicon datasets from past IODP expeditions. These controls included various lubricants used on board, drilling fluids, seawater, DNA extraction blanks and PCR blanks. The order *Burkholderiales* dominated most of the very low biomass samples, including negative controls, indicating the order’s ubiquity and its potential to be overamplified with common 16S rRNA amplification protocols. We amplified the 16S rRNA gene from preserved IODP legacy microbiological core samples and tested their level of contamination using the database. We also looked at published studies that did not sequence negative controls. Our results demonstrate that the type of drilling, amount of manipulation of the sample prior to preservation and sample depth, often associated with biomass, can influence the level of contamination within subsurface samples. This work provides an analysis framework for microbial taxonomic survey studies from low biomass subsurface samples for future scientific ocean drilling expeditions.

## Data Summary

The sequences generated in this study are available in NCBI SRA database under accession numbers SRR29297802–SRR29297810 in the BioProject #PRJNA1120072.

## Introduction

Deep-sea subsurface environments typically have very low microbial biomass [1]. For example, cell abundances within the basaltic rock from the Atlantis Massif (IODP Exp. 357 Atlantis Massif Serpentinization and Life) ranged between 10 and 1000 cells per cm^3^ [2]. Sediments can host between 10^2^ and 10^9^ cells per gramme of sediment and vary based on the sedimentation rate, nutrient availability and biomass, and metabolic rate [3]. Because micro-organisms are ubiquitous, the lower the biomass, the higher the potential for the samples to get contaminated during any step of sample processing, including drilling and transit to the ship (contamination from the drilling fluid, lubricants [4] and seawater [5]), sample preparation (contamination from air and human handling [6]), and even DNA extraction and amplification (contamination from reagents [7]).

Drilling fluids are essential in scientific ocean drilling to cool equipment, control pressure, transport cuttings and stabilize borehole walls. Drilling fluids are a mixture of sepiolite and seawater and thus contain a unique mixture of potential contaminants. Methods have been developed to determine if a sample has been contaminated by drilling fluids. For example, during the International Ocean Drilling Program (IODP) expeditions, samples that are dedicated to microbiological analyses (known as MBIO samples) are tested onboard for contamination by injecting known concentrations of fluorescent microspheres or fluorescent tracers directly into the drilling fluids [8]. The concentration of these volatile compounds is measured using gas chromatography (GC) on multiple negative and positive controls. (i) A sample from the outside of the selected core piece that has been in contact with drilling fluids, seawater and the air. This sample is typically removed when handling the MBIO samples and should be positive. (ii) Chunks from the inside of the same piece after the cores are split with sterile tools. These correspond to the MBIO sample and should be negative. (iii) Small pieces found loose in the core liner. These are considered contamination-positive controls since they have been directly exposed to the drilling fluids and tracer. (iv) Any fluid remaining in the liner. These fluids are also considered a positive control because they should contain a dissolved tracer if the tracer was properly circulating during coring [8]. While these tracers are a great tool to quickly identify samples that have been contaminated by drilling fluids, they are not routinely used, and the samples are still at risk of contamination during subsequent handling and processing.

The IODP core repositories currently hold a large collection of legacy MBIO whole-round core samples from multiple expeditions. These samples have been appropriately preserved and stored for microbiological analyses; that is, they have been processed to remove potential drilling fluid contamination prior to being frozen at −80 °C (wash cores) or have been directly frozen at −80 °C. These samples are available to whoever wants to perform microbiological analyses. They offer a range of contrasting mineralogy, geochemistry and environmental conditions, for the potential of various microbial community structures. The IODP legacy core samples include cores from high-pressure trenches and acidic and low-to-high-temperature hydrothermal systems from divergent and convergent plate margins (available samples listed in Table S1, available in the online Supplementary Material). While some of these core samples are from microbiology-focused expeditions (Table S1), a majority are not. For example, the NanTroSEIZE expeditions, with the goal of better understanding subduction zone earthquakes, allowed the preservation of MBIO samples without having a focus on microbiology. Because most subsurface environments are low biomass, they are at high risk of contamination from air, seawater and handlers, which all have a higher microbial biomass than the core samples. Moreover, since these samples may not have been handled specifically for microbiology purposes (i.e. handlers must wear masks and gloves and work in a sterile environment as soon as possible), it is more likely that contaminants were introduced during collection and processing. Therefore, there is a need to test the level of contamination at the molecular level to determine if these stored samples are suitable for further microbiological analyses by scientists from the IODP community.

Most of the studies that use frozen core samples perform molecular analyses consisting of either extracting total DNA for sequencing (metagenomics) (e.g. [9,10]) or for amplification and sequencing of marker genes such as the 16S and 18S rRNA genes (rRNA) (e.g. [11,12]). The primers used for the amplification of rRNA genes can be biassed towards some taxa while missing others [13]. Contaminants can be identified through the use of negative controls [14], such as substituting molecular-grade water for sample when extracting DNA or performing PCR reactions (DNA extraction blanks and PCR blanks) and molecular analysis of other potential sources of contamination, i.e. drilling fluids or laboratory air. Here, in addition to sequencing our own DNA extraction and PCR blanks, we compared the controls from other studies to build a library of common contaminants associated with the IODP and scientific ocean drilling programmes at large. We tested the level of contamination of a few preserved MBIO samples. Using our database, we also assessed the potential for contamination in previously published datasets that omitted negative controls in their analyses. We asked the following questions: (i) How much contamination can be found in the IODP sediment and basement samples?; (ii) What is the potential source of contamination?; and (iii) Are preserved IODP samples collected on previous IODP expeditions suitable to use for microbiological studies?

## Methods

### Literature review and sequence download

We searched for published studies in the Web of Science database using the terms ‘IODP’ and ‘16S rRNA gene’ (Table S2). A total of 60 papers were identified. To be included in the below analyses, the studies needed to fulfil the following criteria: (i) have any type of ‘controls’ to assess the level of contamination (e.g. seawater, drilling fluids, extraction blanks and PCR blanks), (ii) the 16S rRNA sequences needed to have been generated using Illumina technology, (iii) the sequences had to be from natural samples and not from enrichments to reflect the natural community and (iv) the raw reads had to be available in the NCBI SRA database. Unfortunately, the Census of Deep Life project [14], which contained many potential datasets of interest, utilized Roche 454 Pyrosequencing technology, which had a high error rate and is now obsolete [15], making it unsuitable for the analysis of amplicon sequence variants (ASVs) [16]. Four studies [11,12, 17, 18] fulfilled all four criteria.

### Sequence processing and analysis

The raw sequence datasets from the four selected studies were downloaded from the SRA database (accession numbers available in Table S2). The downloaded raw sequences were analysed using the DADA2 workflow for Big Data, Paired-end edition [19]. Prior to filtering, the datasets were divided into three groups based on the length and overall quality of the PCR products: (1) ~150–200 bp (datasets DRR179383, DRR179384, DRR179385, DRR179386, DRR179387, DRR179388, DRR179389 and DRR179390), (2) ~250 bp (datasets DRR179392, DRR181532, SRR10218173, SRR10218174, SRR10218175, SRR10218176 and SRR10218177) and (3) ~300 bp (datasets DRR179391, DRR181232, DRR181233, DRR181234, DRR181235, DRR181236, DRR181237, DRR181238, DRR181239, DRR181240, DRR181241, DRR181533, DRR181534, DRR181544, DRR181545, DRR181546, DRR181547, DRR181548, DRR181549, DRR181550, DRR181551, DRR181552, DRR181553, DRR181844, DRR181845 and DRR181846). Each group was filtered individually, with minor adjustments for each group. No trimming was necessary for the datasets in group 1 as they maintained a high quality over the entire length of the sequences. For the datasets in group 2, the forward and reverse reads were trimmed to 240 and 150 bp, respectively. For the datasets in group 3, both the forward and reverse reads were trimmed to 250 bp. Filtering and inference of the sequence variants were done for each group before merging the runs, chimaera removal and taxonomic assignment was completed for the entire dataset. ASV taxonomic assignment was completed using the Silva v138.1 training dataset with species annotations. The results that are presented are only for the samples that were labelled as ‘controls’ (e.g. background seawater, drilling fluids and blanks).

To test for contamination in previous studies, the raw sequence datasets were downloaded from the SRA database and analysed following the DADA2 pipeline. The ASVs were labelled as ‘potential contaminants’ (among the 25 most abundant orders identified in blanks and drilling controls), ‘seawater influenced’ (among the 25 most abundant orders identified in the seawater samples) or ‘endemic’ (not found in controls) based on their taxonomic assignment.

### PCR amplification, sequencing and analysis

Six samples were received from the Kochi Repository (Table 1). These samples were thawed in an anaerobic chamber. Once thawed, the centre of the core, or the material presumably uncontaminated, was recovered using sterile spatulas and cutoff syringes. Total DNA was extracted from 10 g of material using the DNeasy PowerMax Soil Kit (Qiagen). The extracted DNA was stored at −20 °C until PCR amplification. Each time DNA extraction was carried out, a DNA extraction blank, using 10 ml of 0.2 µm filtered sterile ultrapure water, was also extracted to identify possible contamination from the ambient laboratory and kit reagents.

**Table 1.** List of the samples received from the Kochi Core Center that were tested in this study.Drilling strategies: H, advanced piston coring; X, extended core barrel; R, rotary core barrel.

Expedition	Site	Hole	Core	Drilling technology	Section	Depth	Type of sample
331	C0013	D	1	H	1	3.52–3.57	Plug
331	C0013	E	1	H	2	1.09–1.24	Plug
333	C0011	D	3	H	6	45.295–45.395	Whole round
333	C0011	D	17	H	7	155.955–156.055	Whole round
333	C0011	D	38	X	2	285.23–285.32	Whole round
370	C0023	A	83	R	4	867.06–867.16	Whole round

The Earth Microbiome Project PCR primers 515F-Y (5′-GTGYCAGCMGCCGCGGTAA-3′) and 926R (5′-CCGYCAATTYMTTTRAGTTT-3′) were used to target the V4–V5 region of the 16S rRNA gene [20,21]. Thermal cycling was performed under the following conditions: initial preheating for 3 min at 94 °C; 35 cycles of denaturation at 94 °C for 1 min, annealing at 50 °C for 1 min and extension at 72 °C for 1 min 45 s; and final extension at 72 °C for 10 min. PCR reactions were completed in triplicate. A PCR blank containing all the same reagents and no template was also generated. Each reaction was visualized with an agarose gel electrophoresis. The triplicate products were then pooled and cleaned using the Select-a-Size DNA Clean and Concentrator Kit (Zymo Research), selecting fragments >150 bp to remove primer dimers. Amplicons (100–200 ng) were sent to the Texas A and M AgriLife Bioinformatics and Genomics facility for sequencing with Illumina MiSeq with 250 bp paired ends.

The PCR amplicons were analysed using the DADA2 pipeline according to the tutorial 1.16 [19], and the ASVs were labelled as ‘potential contaminants’, ‘seawater influenced’ or ‘endemic’ (not found in controls) as described above.

## Results

A total of 313 samples from 4 studies were identified as negative controls and used for further analyses. Controls were grouped based on their source type, regardless of the study: (I) *scientific ocean drilling-associated contamination*, including swabs of drill-related equipment, oils, lubricants and paints used on board; (II) *laboratory contaminants*, found in molecular reagents and ambient laboratory air; and (III) *seawater and drilling fluids*, which are associated with drilling fluids being a mix of seawater and sepiolite.

**Fig. 1.**
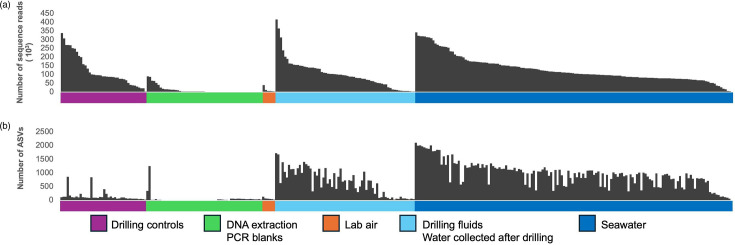
(a) Total number of reads in each control sample. The samples were sorted by their number of total reads within each type of contamination control. (**b**) Total number of ASVs in each sample. The samples were sorted as in (**a**), highlighting that more sequence reads do not necessarily equal to an increase in ASVs.

On average, the datasets had between 0 and 413 923 reads, with an average of 120 814, 9780 and 119 416 reads that passed quality control (filtering, merging and chimaera removal) for control groups I, II and III, respectively (Fig. 1). A total of 30 960 ASVs were identified within 284 datasets (Fig. 1). For 29 samples, all of which were DNA extraction or PCR blanks, no ASV was generated. In general, the number of sequenced reads did not impact the number of identified ASVs; i.e. the samples with fewer reads did not generate fewer ASVs or had lower richness. Overall, there were fewer ASVs within the negative controls than in seawater-associated samples, including drilling mud (Fig. 1b).

### Contamination from drilling equipment and laboratory contaminants

**Fig. 2.**
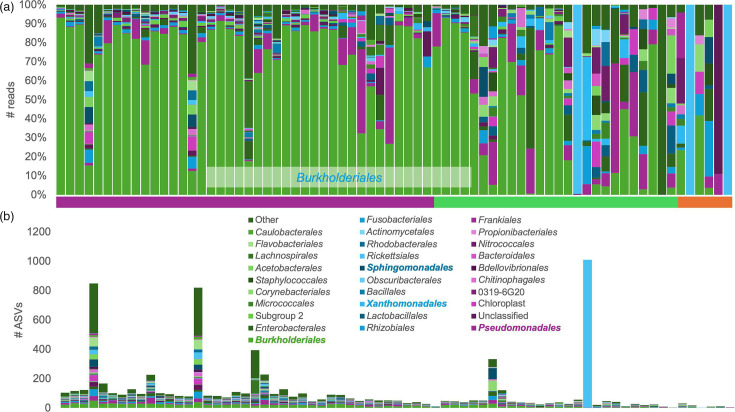
Taxonomic assignment of the ASVs identified in laboratory controls shown as relative abundance (**a**) and the number of ASVs assigned to each order (**b**). The colours at the base of (**a**) represent the contamination sources drilling fluids (purple), DNA extraction and PCR blanks (green) and lab air (orange). The order *Burkholderiales* dominated most samples in terms of the number of sequences, but not in terms of unique ASVs.

Within control types I and II (total of 100 samples), 5096 ASVs were identified. Among these, 4773 ASVs were sequenced more than 5 times, i.e. consisted of a total of 5 reads or more within a single sample or among multiple samples. Of these, 808 ASVs were found in more than 1 sample (Table S3). The 25 ASVs that occurred the most are presented in Table 2. The two most abundant ASVs occurred in 54 and 52 samples, respectively, and were both identified as belonging to the genus *Ralstonia* (class *Gammaproteobacteria*, order *Burkholderiales*, family *Burkholderiaceae*). Eleven other ASVs were part of the order *Burkholderiales*. *Burkholderiales* was the dominant order in most of the samples from control groups I and II, representing 0.6–93.2% (median=73.3%) of the relative abundance (Fig. 2). Other abundant orders included *Pseudomonadales*, *Xanthomonadales* and *Sphingomonadales* (Table 3). The high prevalence of these ubiquitous orders in samples that should not contain any sequences (DNA extraction and PCR blanks) or have very little biomass (laboratory air, greases and lubricants) strongly suggests that they are likely the result of contamination when found in subsurface samples. In the controls, most ASVs were not evenly distributed as the relative abundance of specific ASVs tended to be dominated by certain taxonomic orders (Fig. 2). This was especially true for *Burkholderiales*, which made up a large proportion of all the reads sequenced but made up a smaller percentage of the unique ASVs generated (Fig. 2b). This suggests that *Burkholderiales* species were very abundant in the samples or that the PCR primers are biassed for *Burkholderiales*, which would lead to *Burkholderiales* sequences being preferentially overamplified during the PCR amplification step. Preferential amplification of taxa has been observed before and can be influenced by the microbial community and type of environment [20,22]. The other ASVs were generally proportionately represented in the sequence relative abundance and ASV distribution (Fig. 2).

**Table 2.** List of the 30 most common laboratory (control groups I and II) and seawater (control group III) contaminants, at the order level

Laboratory contaminant	Seawater contaminant
0319-6G20 (*Deltaproteobacteria*)	*Actinomarinales*
*Acetobacterales*	Burkholderiales*
*Actinomycetales*	Chloroplast*
*Bacillales*	*Defluviicoccales*
*Bacteroidales*	*Ectothiorhodospirales*
*Bdellovibrionales*	*Enterobacterales*
Burkholderiales*	Flavobacteriales*
*Caulobacterales*	HOC36 (*Pseudomonadota*)
*Chitinophagales*	*Lactobacillales*
Chloroplast*	Marine Group II
*Corynebacteriales*	Marine Group III
*Enterobacterales*	Micrococcales*
Flavobacteriales*	*Microtrichales*
*Frankiales*	*Nitrosopumilales*
*Fusobacteriales*	*Nitrospinales*
*Lachnospirales*	*Opitutales*
*Lactobacillales*	*Phycisphaerales*
Micrococcales*	Pseudomonadales*
*Nitrococcales*	*Puniceispirillales*
*Obscuribacterales*	Rhodobacterales*
*Propionibacteriales*	*Rhodospirillales*
Pseudomonadales*	Rickettsiales*
*Rhizobiales*	SAR11 clade
Rhodobacterales*	SAR202 clade
Rickettsiales*	Staphylococcales*
*Sphingomonadales*	*Synechococcales*
Staphylococcales*	UBA10353 marine group
Subgroup 2 (*Acidobacteriae*)	*Vicinamibacterales*
*Xanthomonadales*	*Woesearchaeales*

* Orders found in both groups of contaminants, therefore ubiquitous micro-organisms.

**Table 3.** Taxonomic assignment of the 25 ASVs that occurred in most samples from control groups I and II

Class	Order	Family	Genus	ASV occurrence
*Gammaproteobacteria*	*Burkholderiales*	*Burkholderiaceae*	*Ralstonia*	54
*Gammaproteobacteria*	*Burkholderiales*	*Burkholderiaceae*	*Ralstonia*	52
*Gammaproteobacteria*	*Burkholderiales*	*Burkholderiaceae*	*Burkholderia-Caballeronia-Paraburkholderia*	47
*Gammaproteobacteria*	*Burkholderiales*	*Burkholderiaceae*	*Burkholderia-Caballeronia-Paraburkholderia*	46
*Gammaproteobacteria*	*Burkholderiales*	*Comamonadaceae*	*Paucibacter*	45
*Gammaproteobacteria*	*Xanthomonadales*	*Xanthomonadaceae*	*Stenotrophomonas*	44
*Gammaproteobacteria*	*Burkholderiales*	*Burkholderiaceae*	*Burkholderia-Caballeronia-Paraburkholderia*	41
*Acidobacteriae*	Subgroup 2	na	na	39
*Gammaproteobacteria*	*Burkholderiales*	*Alcaligenaceae*	*Achromobacter*	39
*Gammaproteobacteria*	*Burkholderiales*	*Burkholderiaceae*	*Burkholderia-Caballeronia-Paraburkholderia*	37
*Bacilli*	*Staphylococcales*	*Staphylococcaceae*	*Staphylococcus*	37
*Oligoflexia*	0319-6G20	na	na	34
*Gammaproteobacteria*	*Burkholderiales*	*Comamonadaceae*	*Aquabacterium*	33
BD7-11	na	na	na	33
*Alphaproteobacteria*	*Rhizobiales*	*Xanthobacteraceae*	na	32
*Bdellovibrionia*	*Bdellovibrionales*	*Bdellovibrionaceae*	*Bdellovibrio*	31
*Acidobacteriae*	Subgroup 2	na	na	29
*Gammaproteobacteria*	*Burkholderiales*	*Burkholderiaceae*	*Burkholderia-Caballeronia-Paraburkholderia*	28
*Alphaproteobacteria*	*Reyranellales*	*Reyranellaceae*	*Reyranella*	28
*Gammaproteobacteria*	*Burkholderiales*	*Rhodocyclaceae*	*Denitratisoma*	27
*Alphaproteobacteria*	*Rhizobiales*	*Rhizobiaceae*	*Aminobacter*	27
*Gammaproteobacteria*	*Pseudomonadales*	*Moraxellaceae*	*Acinetobacter*	25
*Holophagae*	*Holophagales*	*Holophagaceae*	na	25
na	na	na	na	25
*Gammaproteobacteria*	*Burkholderiales*	*Comamonadaceae*	*Delftia*	25

nanot applicable

### Contamination from seawater and drilling mud

**Fig. 3.**
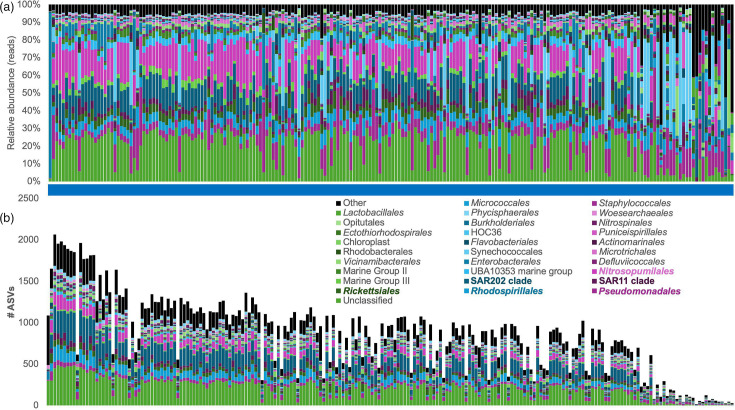
Taxonomic assignment of ASVs identified in seawater controls shown as relative abundance (**a**) and the number of ASVs assigned to each order (**b**).

In contrast to the low biomass samples, which were dominated by *Burkholderiales* and had an uneven distribution of taxa, the microbial composition of the seawater samples was very similar across samples, with a dominance of *Pseudomonadales*, *Rhodospirillales*, *Rickettsiales*, SAR202 clade and *Nitrosopumilales*. Surface samples were also dominated by the cyanobacteria *Synechococcales*. Moreover, the distribution of ASVs generally reflected the distribution of the sequences, except for the cyanobacteria *Synechococcales*, which, similar to *Rhodobacterales*, displayed higher relative abundances of the sequences than unique ASVs identified (Fig. 3).

### Building and testing a database of possible contaminants

**Fig. 4.**
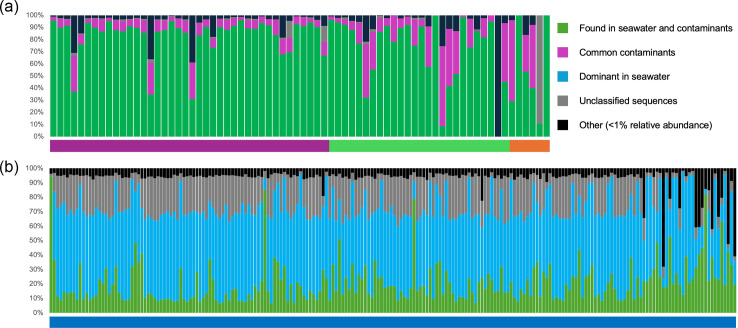
Fraction of the sequences that are represented by the most dominant orders in the hereby assembled database identified in the negative controls (**a**) and seawater (**b**). The colours at the base of the histograms represent the contamination sources, either drilling fluids (purple), DNA extraction and PCR blanks (green), lab air (orange) or seawater (blue).

Based on our review of all the contaminants, we made a compilation of the 30 most abundant orders identified in the drilling and laboratory contaminants (control groups I and II) and the seawater (control group III) (Table 2). Using a consistency pattern approach as commonly used in microbiome research [23] (Fig. 4), the identified contaminants represented >95% of all reads in 53 of the 70 samples. These orders were all present at a relative abundance higher than 1% of the total number of reads found in all samples. A majority of the contaminants were found in both contamination controls and seawater. The orders found solely in control groups I and II were identified as ‘most likely contaminants’ since they were found in samples that should have no micro-organisms or environments that were in contact with the samples but are naturally far from the subsurface. The orders found in control group III (seawater) were identified as ‘seawater contaminants or seawater influenced’. Given the fluid flow in subsurface environments, it is possible that micro-organisms are transported via these fluids and are truly present in the sample. Alternatively, they could also consist of contamination coming from the drilling fluids.

Table S3 presents a list of all the ASVs identified and their prevalence in each sample. Because we used amplicons from multiple studies that did not always sequence the same region, we had to use a taxonomic cutoff rather than an ASV sequence cutoff. We have decided to present our analyses at the order level. While the 16S rRNA gene is ~1 550 bp, the products generated using degenerate primers focus on a single region of the gene, typically the V4 region (~254 bp), V4–V5 (~412 bp) and V3–V4 region (443 bp). Complete gene sequences can allow taxonomic assignment down to the species level, but the loss of information in the partial genes does not allow for assignment with confidence at lower taxonomic levels. Because a majority of micro-organisms in the environmental and contamination control samples belong to the *Proteobacteria* phylum, more specifically to the *Alphaproteobacteria* class, we opted for the order level, which allowed for confident taxonomic assignment and separation of likely contaminants from likely endemic micro-organisms.

We performed 16S rRNA gene sequencing of the V4–V5 region on six sediment samples from the Japan Trench collected during IODP Exp. 331 Deep Hot Biosphere, IODP Exp. 333 NanTroSEIZE Stage 2: Subduction Inputs 2 and Heat Flow and IODP Exp. 370 Temperature Limit of the Deep Biosphere off Muroto. Some of these samples were collected by microbiologists (i.e. IODP Exp. 331 and 333), while others were collected by other members of the science party. The samples were collected using different drilling strategies: rotary core barrel (R), advanced piston coring (H) or extended core barrel (X). One sample was also a wash core (W); i.e. the outside of the core that had touched the core liner had been removed sterilely before freezing the sample (Table 1). Two samples consisted of 10 cc plugs, which are typically sampled using a sterile cutoff syringe before freezing.

During the processing and amplification of the six MBIO-preserved samples, we generated our own control samples with DNA extraction and PCR blanks. Every order identified in our database was also identified in our negative control. When considering the orders present at >1% relative abundance in all samples, there were no more potential contaminants remaining in the samples.

**Fig. 5.**
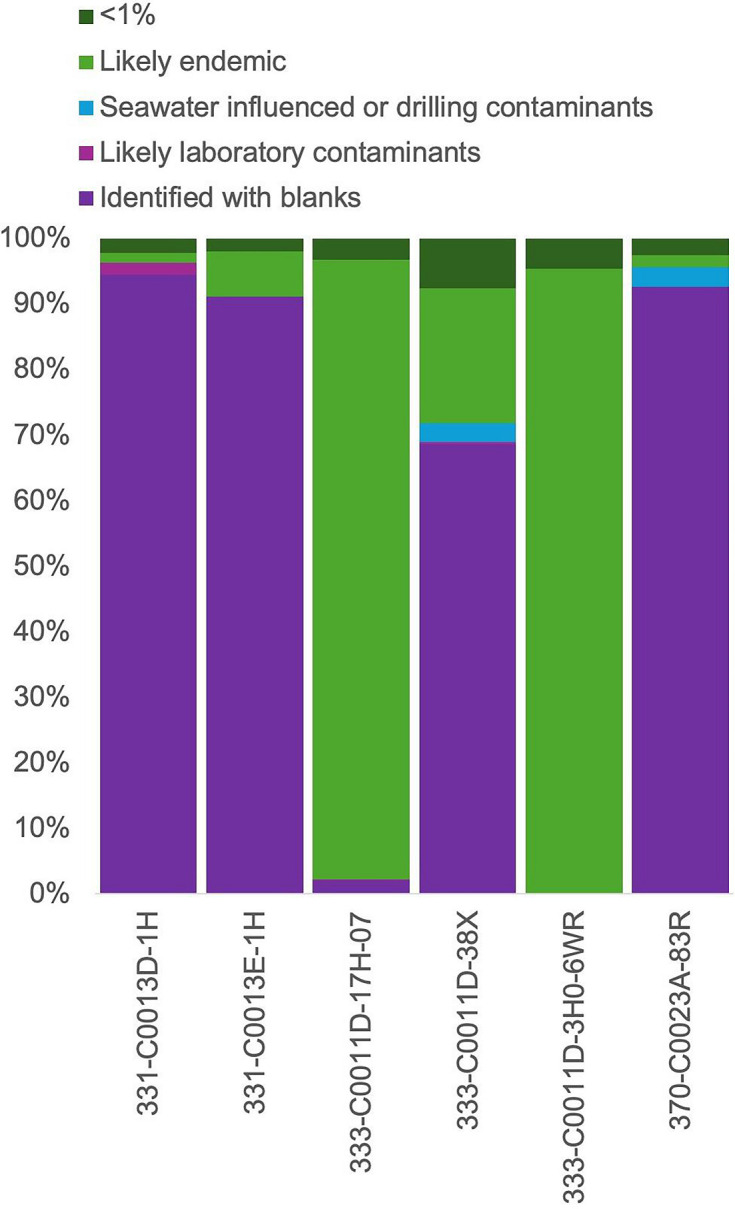
Evaluation of the quality of six IODP Legacy preserved samples, indicating that the drilling strategy, excessive manipulation of the sample and initial concentration of biomass influence the amount of contamination. ‘<1% relative abundance’ represents the orders that were present at less than 1% relative abundance.

When using stringent settings, i.e. flagging of any ASV belonging to the 30 orders, the samples contained between 0.03 and 94.5% contamination (Fig. 5). Most of the contamination was identified within the laboratory contaminants, as opposed to seawater. Two samples had little to no contamination, the wash core (333-C0011D017H-07) and the shallow rotary core barrel sample (333-C0011D-3H0-6WR). The most contaminated samples consisted of 10 cc plugs (331-C0013D-1H and 331-C0013E-1H) and a deep whole round sample collected using a rotary core barrel (370-C0023A-83R). These results indicate that contamination is more likely to occur (i) when the sample is heavily manipulated, such as is the case of plugs; (ii) when the coring strategy is disturbing sample integrity (such is the case with rotary core barrel); and (iii) when the biomass is lower, for example, in deeper samples.

**Fig. 6.**
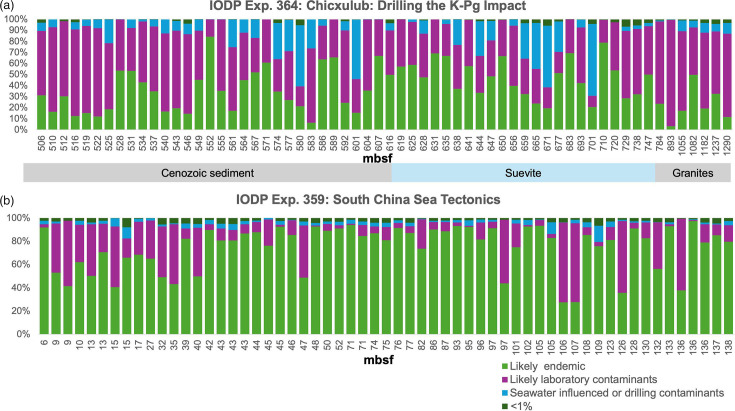
Evaluation of the potential contamination in two published IODP expeditions that did not include blanks in the publication. The laboratory and seawater contaminants included the 30 classes identified as the most dominant ones in Figs2  3, respectively. The samples are sorted by depth measured as metres below seafloor (mbsf). ‘<1%’ represents the orders that were present at less than 1% relative abundance.

We tested our database on two published IODP studies that did not have controls to evaluate the level of possible contamination (Fig. 6). The same stringency as was used with our negative controls was applied to the samples from IODP Exp. 364: Chicxulub: Drilling the K-Pg Impact Crater [24] and IODP Exp. 359: South China Sea Tectonics [25]. During IODP Exp. 364, a single site was drilled using a rotary core barrel (R) from a platform (not a research vessel). IODP Exp. 359 drilled at eight sites using a combination of advanced piston coring (H), extended core barrel (X) and rotary core barrel (R). As expected, the amount of potential contamination, ranging from 15.6 to 96.8% (median=64.9%), is higher in the IODP Exp. 364 samples due to the use of more disruptive drilling technologies. However, there was no relationship between the type of drilling technology and the type of material sampled (sediment, suevite or granites) (Fig. 6). The samples from IODP Exp. 364 also had more potential contamination from seawater, which can be caused by the use of drilling fluids. Among the IODP 359 samples, a total of 22 samples (or 37%) consisted of >20% contamination. However, all the samples had >30% likely endemic sequences, and 52/60 samples had <50% likely contaminants. Those samples were also shallower.

## Discussion

The study of the microbes inhabiting the deep subsurface is challenged by the low biomass encountered in these environments, especially in environments with low sedimentation rates [3]. The low biomass, combined with the presence of molecular inhibitors, can prevent the extraction of high quantities of quality genomic material [26], as well as inhibit PCR amplification, library preparation and sequencing. Large quantities of DNA (in the hundreds of nanogrammes) are required to determine the community composition and genomic potential of the deep biosphere communities using metagenomics. Given the difficulty in getting large concentrations of high-quality DNA and the high cost of metagenomic sequencing, the first step to characterize the microbial community composition in samples from unexplored environments usually consists of the identification of the microbiome using a marker gene such as the universally encoded rRNA gene (rRNA) [27,28]. Given that micro-organisms are ubiquitous and often found in higher abundance on our skin and even in ambient air than in deep-sea subsurface samples, the risk of overamplifying contaminants greatly increases when using PCR amplification of the 16S rRNA gene to characterize the microbial community composition. It is therefore of utmost importance to perform the necessary controls to assess the potential and origin of contaminants [1,14]. While controls should be routinely performed in the laboratory, issues can arise and mistakes can be made, leading to controls missing in the analysis. Here, we propose a simple, straightforward way to identify and exclude common contaminants from subsurface samples collected using scientific ocean drilling.

The first scientific ocean drilling expedition dedicated to study subseafloor micro-organisms was the Ocean Drilling Project (ODP) Leg 201: Deep Peru Biosphere in 2003 [29]. Other early expeditions included the International Ocean Drilling Program (IODP) Exp. 329: South Pacific Gyre Subseafloor life [30]. In the current phase of the IODP, IODP Exp. 337 Deep Coalbed Biosphere off Shimokita was the first expedition [12]. Since 2015, at least six expeditions had at least one objective dedicated to the study of the deep biosphere [Exp. 357: Atlantis Massif Serpentinization and Life [18,31], Exp. 370: Nankai Trough Temperature Limit [32], 366: Mariana Convergent Margin and S. Chamorro Seamount [33], 376: Brothers Arc Flux (Campbell *et al*., in prep.), 385: Guaymas Basin Tectonics and Biosphere [34] and 390/393: South Atlantic Transects]. The collaboration between the IODP and microbiologists has allowed the development of techniques onboard that allow the determination of contamination by drilling fluids through the injection of fluorescent microspheres or tracers in the drilling fluids (reviewed in [8]). These are practical to readily determine the suitability of samples onboard when routinely used, but their high cost prevents their use for every drilled hole, and they are only a measure of drilling contamination. In this study, we showed that drilling contamination depends on the drilling method used. Indeed, more destructive methods, such as a rotary core barrel or extended core barrel, can lead to increased microbial contamination within the samples. When collecting microbiological samples using these drilling methods, it becomes even more important to aseptically remove any parts of the core that came in contact with drilling fluids to limit or remove contamination, as was done for the wash core.

We built a database of the 30 most common orders identified in the laboratory and seawater/drilling controls sequenced during the analysis of the IODP samples. We showed that some contaminant taxa, such as *Burkholderiales*, are dominating low biomass samples. *Burkholderiales* members can therefore be overamplified during the PCR step, leading to these taxa dominating the read counts, but not the ASV counts. Using our database, we were able to identify the vast majority, if not all, of the contaminants for taxa present at a relative abundance >1% in six IODP Legacy samples preserved for microbiological studies (MBIO). While this database should not be used in lieu of controls, it could be used as a complement to controls, or when controls are unavailable [i.e. preserved MBIO IODP core samples from non-microbiology-focused studies (Table S1)], to determine which taxa are potential contaminants from handling and molecular reagents, but also seawater infiltration or drilling fluids. Note that to provide informative information about the microbial community composition, we recommend describing taxonomy at the order level, which allows significant taxonomic assignment while remaining informative.

While this database indicates that micro-organisms belonging to the order *Burkholderiales*, for example, are likely a sign of contamination, it remains possible that these micro-organisms are endemic to the subsurface due to their ubiquitous nature. For example, ubiquitous actinobacterial sequences were found to dominate basalt samples from the Juan de Fuca Ridge [5]. Although it was not possible to confirm that these sequences were the result of contamination, phylogenetic analyses of the sequences along with other actinobacterial sequences from the subsurface, soil, isolates and other environments suggested that they were likely contaminants. Indeed, the basalt sequences were closely related to the isolates and sequences found in the human microbiome [5]. This could also be due to the analyses relying on sequences available in the databases. While there are ongoing efforts to increase the number of sequences coming from understudied environments, the databases are largely biassed towards sequences from organisms with a societal impact, e.g. human-associated, plant and livestock diseases. Moreover, a majority of the 16S rRNA sequences that are available are partial sequences (e.g. V4 and V4–V5), and there remain large gaps in microbial taxonomy. Due to the biassed nature of PCR amplification, we recommend using a stringent approach for the removal of contaminants and removing anything that can be suspected of contamination, acknowledging that some ubiquitous taxa may be misinterpreted as contaminants.

We have tested our database on the IODP Legacy samples, as well as on published datasets. After manipulation, the IODP MBIO Legacy samples showed up to 95% contamination when using 16S rRNA amplicon sequencing. The amount of contamination from published datasets varied based on the drilling strategy. As we drill deeper in the seafloor, sediments get more compacted, leading to the need to use more disturbing drilling technologies. It has also been shown that in general, cell abundance, or microbial biomass, decreases with depth below seafloor [3,12], which can increase the contaminant to endemic micro-organism ratio. We demonstrated that excessive manipulation of the sample prior to freezing, such as the preparation of plugs, can lead to increased contamination as samples 331-C0013D-1H and 331-C0013E-1H were the most contaminated.

## Conclusions and recommendations

We propose a few recommendations when sampling and analysing low biomass subseafloor samples using molecular methods. We suggest that sediment samples to be used for microbiological studies be cut straight from the catwalk using sterile tools such as autoclaved spatulas, sealed with sterile caps sprayed with 70% (v/v) ethyl alcohol and placed in a sealed bag before immediately freezing at −80 °C. All personnel should be wearing a mask and gloves sprayed with 70% (v/v) ethyl alcohol when handling microbiological samples. When enough samples and funds are available, it would be best to limit the overamplification of samples by using less biassed molecular techniques, such as metagenomics, or even single-cell genomics [35], although these methods also have biases due to the lack of representative reference genomes in the databases that make contaminant identification more challenging. A combination of 16S rRNA gene sequencing and omics is recommended when possible. When performing surveys using the 16S rRNA marker gene, it is important to perform and sequence controls at each step of the process (i.e. DNA extraction and PCR amplification). We recommend performing duplicates and triplicates for each sample to account for the variability within environmental samples. Finally, when analysing the data, the potential contaminants should be labelled as such or removed from the final analyses. Our recommendations could also be used for researchers who are dealing with low biomass samples that necessitate thorough negative controls.

## supplementary material

10.1099/acmi.0.000865.v3 Uncited Table S1.

10.1099/acmi.0.000865.v3 Uncited Table S2.

10.1099/acmi.0.000865.v3 Uncited Table S3.

## Abbreviations

ASV: amplicon sequence variant
IODP: International Ocean Discovery Program
